# Supplementary material for: Quantum decoherence of dark pulses in optical microresonators
Source: Nat Commun. 2023 Mar 31;14:1802. doi: 10.1038/s41467-023-37475-z (PMC10066214; doi:10.1038/s41467-023-37475-z)
Supplement: Supplementary file 1 — Supplementary Information [file 41467_2023_37475_MOESM1_ESM.pdf]

Supplementary information: Quantum decoherence of dark pulses in optical microresonators

Chenghao Lao[1*], Xing Jin[1*], Lin Chang[2*], Heming Wang[3], Zhe Lv[1], Weiqiang Xie[3,5], Haowen Shu[2], Xingjun Wang[2], John E. Bowers[3†], and Qi-Fan Yang[1,4†]
[1]State Key Laboratory for Artificial Microstructure and Mesoscopic Physics and Frontiers Science Center for Nano-optoelectronics, School of Physics, Peking University, Beijing 100871, China
[2]State Key Laboratory of Advanced Optical Communications System and Networks, School of Electronics, Peking University, Beijing, 100871, China
[3]Department of Electrical and Computer Engineering, University of California, Santa Barbara, CA 93106, USA
[4]Collaborative Innovation Center of Extreme Optics, Shanxi University, 030006, Taiyuan, China
[5]Present address: State Key Lab of Advanced Optical Communication Systems and Networks, Department of Electronic Engineering, Shanghai Jiao Tong University, Shanghai 200240, China
[*]These authors contributed equally to this work.
[†]Corresponding author: bowers@ece.ucsb.edu, leonardoyoung@pku.edu.cn

I. THEORETICAL AND NUMERICAL ANALYSIS

A. Impact of avoided-mode-crossings

While dark pulses are predicted to exist in an ideal microresonator, their excitation using a tunable continuous-wave laser is rather difficult. In practice, pumping at avoided-mode-crossings(AMX) has been widely adopted to ease excitation. However, it is arguable whether dark pulses generated using the two protocols belong to the same class. In this section, we compare the theoretical formulae that describe the formation of Kerr frequency combs in microresonators with or without AMX. The normalized Lugiato-Lefever equation (LLE) in an ideal microresonator is written as

$$\frac{\partial\psi(\phi,\tau)}{\partial\tau} = id_2\frac{\partial^2\psi}{\partial\phi^2} + i|\psi|^2\psi - (1+i\zeta)\psi + f, \tag{S1}$$

where $\psi(\tau,\phi)$ represents the intracavity waveform at time τ and angular position ϕ, d_2 is the normalized dispersion, ζ is the normalized detuning, and f is the normalized pump term. It is equivalent to the coupled-mode formalism that describes the evolution of comb lines in the spectral domain[1], which is given by

$$\frac{\partial a_\mu}{\partial\tau} = -[1 + i\zeta + id_2\mu^2]a_\mu + \delta_{0\mu}f + i\sum_{\mu_1,\mu_2} a_{\mu_1}a_{\mu_2}a^*_{\mu_1+\mu_2-\mu}. \tag{S2}$$

Here a_μ denotes the field in the $\mu_{\rm th}$ mode and δ_{mn} is the Kronecker delta. On account of AMX, the pump mode is shifted by the amount of Δ, and the modified coupled-mode equations yield

$$\frac{\partial a_\mu}{\partial\tau} = -[1 + i\zeta + id_2\mu^2 - i\Delta\delta_{0\mu}]a_\mu + \delta_{0\mu}f + i\sum_{\mu_1,\mu_2} a_{\mu_1}a_{\mu_2}a^*_{\mu_1+\mu_2-\mu}. \tag{S3}$$

By discrete Fourier transform (DFT), we have the time-domain representation of the coherently-driven Kerr microresonator, given by

$$\frac{\partial\psi(\phi,\tau)}{\partial\tau} = id_2\frac{\partial^2\psi}{\partial\phi^2} + i|\psi|^2\psi - (1+i\zeta)\psi + f_{\rm eff}. \tag{S4}$$

$$f_{\rm eff} = f + i\Delta a_o. \tag{S5}$$

The effective pump term $f_{\rm eff}$ can also be express as $f_{\rm eff} = f + i\Delta\overline{\psi}$. Remarkably, equation S4 has the exact form of a standard LLE (Eq. S1), suggesting that the frequency shift of the pump mode may not change the type of generated

microcombs. Indeed, for a stable dark pulse, a_o remains invariant over time, and we can thus match the two pump terms to realize a pair of identical equations.

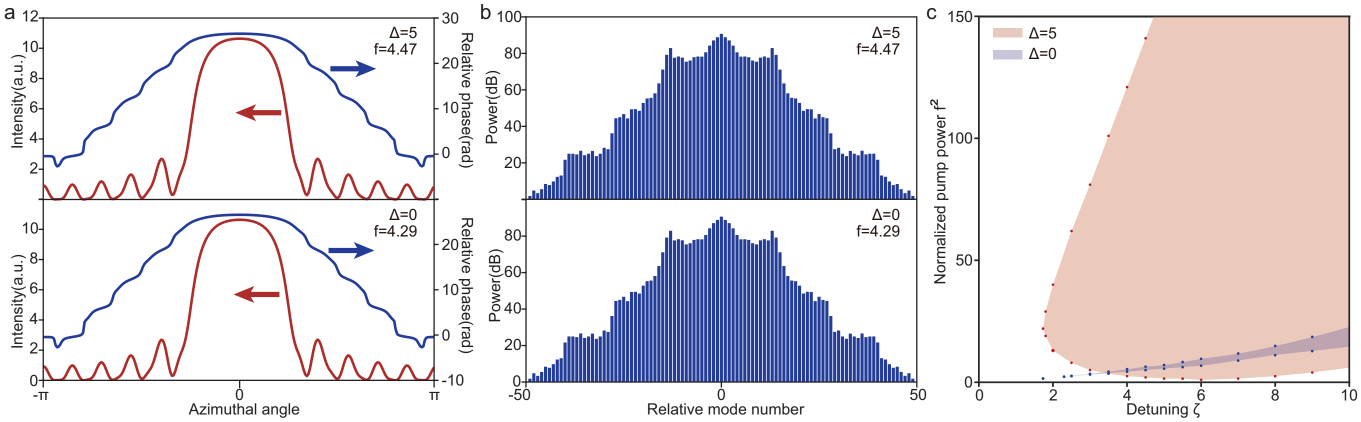

FIG. S1: **Dark pulses in microresonators w/ and w/o AMX at the pump mode. a,** Numerically simulated temporal profiles and phases of dark pulses with $\Delta = 0$ and $\Delta = 5$. The corresponding pump parameters are indicated in the figure. Other parameters used in the simulations are $d_2 = -0.05$ and $\zeta = 10$.**b,** Optical spectra of dark pulses in accordance with **a**. **c,** Phase diagram showing the existence range of dark pulses for $\Delta = 0$ (blue) and $\Delta = 5$ (red).

Numerical simulations further validate the equivalence of the two scenarios. First, a stable dark pulse solution is found using Eq. S4, from which the effective pump term is derived. It is then substituted into Eq. S1 and we can solve for a dark pulse accordingly. Fig. S1 manifests that these dark pulses are identical in spectral and temporal domains. However, the phase diagram shown in Fig. S1c explicitly points out that the existence range of dark pulses can be substantially extended if the resonant frequency of the pump mode is red-shifted. For the sake of simplicity, AMX is not included in analytical studies in this work.

B. Protocols of numerical simulation

The simulation of quantum timing jitter is based on the stochastic LLE (Eq. 1-2 in the main text). We begin with the discretization of the Langevin force[2]

$$\int_0^\infty d\tau \int_{-\pi}^{\pi} \langle \epsilon(\phi,\tau)\epsilon^*(\phi',\tau')\rangle d\phi \approx \sum_{i=1}^{\infty}\sum_{m=1}^{N} < \epsilon'(i\Delta\phi, m\Delta\tau)\epsilon'^*(j\Delta\phi, n\Delta\tau) > \Delta\tau\Delta\phi, \tag{S6}$$

which gives

$$< \epsilon'(i\Delta\phi, m\Delta\tau)\epsilon'^*(j\Delta\phi, n\Delta\tau) > = \frac{2\hbar\omega_o^2 n_2 D_1}{\kappa n_o A_{eff}\Delta\tau\Delta\phi}\delta_{ij}\delta_{mn}. \tag{S7}$$

Here $\Delta\tau$ and $\Delta\phi$ are the step size of time and angular positions respectively. Therefore, the Langevin force can be introduced in the simulation by setting its amplitude to $\sqrt{\frac{2\hbar\omega_o^2 n_2 D_1}{\kappa n_o A_{eff}\Delta\tau\Delta\phi}}$ and randomizing its phases. Likewise, if the Langevin force is added spectrally, it should follow

$$< \tilde{\epsilon}(\mu, i\Delta\tau)\tilde{\epsilon}^*(\mu', j\Delta\tau) > = \frac{\hbar\omega_o^2 n_2 D_1}{\pi\kappa n_o A_{eff}\Delta\tau}\delta_{\mu\mu'}\delta_{ij}. \tag{S8}$$

The timing jitter is derived from the phase noise of the RF signals synthesized from the microcombs, given by:

$$A_{RF} = e^{iD_1 t}\sum_{\mu=-N/2}^{N/2-1} a_\mu^* a_{\mu+1}. \tag{S9}$$

Here N is the number of comb lines involved in the simulation, and a_μ are the complex amplitude of the μ_{th} comb line relative to the pump. The phase of the RF signal is derived as

$$\varphi_{RF} = \mathrm{Arg}[\sum_{\mu=-N/2}^{N/2-1} a_\mu^* a_{\mu+1}], \tag{S10}$$

with Arg computing the phase angle of a complex number. The manifestation of dark pulse time jitter on the generated RF signal is phase noise. Therefore, the timing jitter is derived from the PSD of the RF phase $S_\varphi(f)$ by

$$S_t(f) = S_\varphi(f)/D_1^2. \tag{S11}$$

C. Semi-analytical modeling of quantum dynamics of dark pulses

In this section, we derive the semi-analytical description of the motions of SWs and the dark pulses under quantum fluctuations. A simplified representation of the dark pulse is given by[3,4]

$$\psi(\phi) = \begin{cases} \rho_L & D+W < |\phi| < \pi \\ \psi_{L0}(\phi) & -(D+W) \leq \phi \leq -W \\ \rho_H & |\phi| < W \\ \psi_{R0}(\phi) & W \leq \phi \leq D+W \end{cases} \tag{S12}$$

where ρ_L and ρ_H are the amplitudes of lower and upper levels of the bistable curve respectively. ψ_{L0} and ψ_{H0} correspond to the left and right SWs. W represents the half-width of the upper level of the dark pulse and D is the width of SW. The motions of SW depend on the pumping conditions[3,4]. Equilibrium is only possible when the pump is at the Maxwell point – a situation when the SWs stop moving. When the pumping term is above the Maxwell point, the SW tends to invade the lower level, otherwise, it invades the upper level. We thus conclude the gain and loss of the left SW, which are essentially zero at equilibrium:

$$\int_{-(D+W)}^{-W} (2\,\mathrm{Re}[f\psi_{L0}^*] - 2|\psi_{L0}|^2)d\phi = 0. \tag{S13}$$

Next we consider the injection of photons with random phase θ_n into the n_{th} mode, and the LLE is perturbed as

$$\frac{\partial\psi(\phi,\tau)}{\partial\tau} = id_2\frac{\partial^2\psi}{\partial\phi^2} + i|\psi|^2\psi - (1+i\zeta)\psi + f + \epsilon_n e^{i(n\phi+\theta_n)}, \tag{S14}$$

Here f and $\epsilon_n e^{i(n\phi+\theta_n)}$ together constitute the total pump term f_{t}. Since $\epsilon_n \ll f$, the total pump term can be simplified as

$$f_{\mathrm{t}} = f + \epsilon_n e^{i(n\phi+\theta_n)} = [f + \epsilon_n \cos(n\phi+\theta_n)]e^{i\epsilon_n \sin(n\phi+\theta_n)/f}. \tag{S15}$$

The LSW experiences a net gain given by

$$\begin{aligned} G_L' &= \int_{-(D+W)}^{-W} (2\,\mathrm{Re}[f_t\psi_{L0}^*] - 2|\psi_{L0}|^2)d\phi, \\ &= \int_{-(D+W)}^{-W} 2\,\mathrm{Re}[\epsilon_n e^{i(n\phi+\theta_n)}\psi_{L0}^*]d\phi. \end{aligned} \tag{S16}$$

Note that the SWs are localized, so for modes that are close to the pump a slowly-varying approximation $nD \ll 1$ is applied in the integral. The net gain now reads

$$G_L' = \epsilon_n \int_{-(D+W)}^{-W} [e^{i(n\phi_L+\theta_n)}\psi_{L0}^* + e^{-i(n\phi_L+\theta_n)}\psi_{L0}]d\phi, \tag{S17}$$

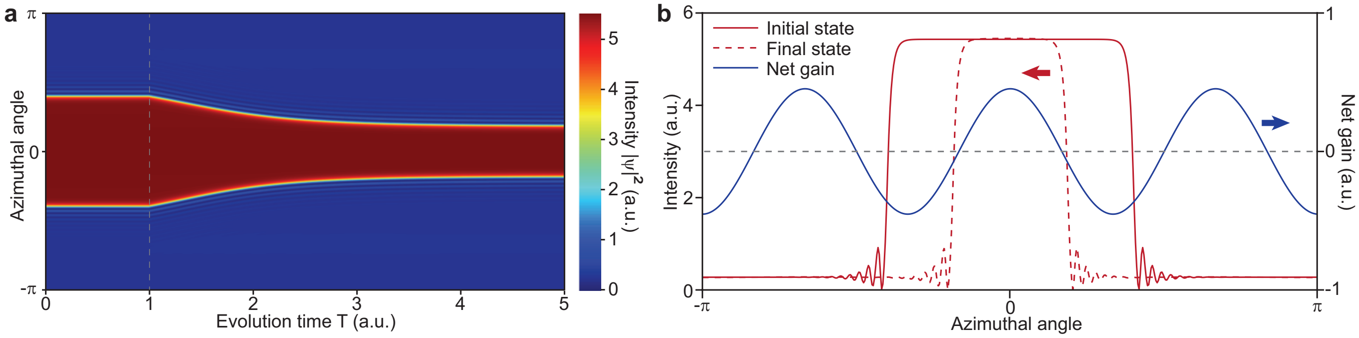

FIG. S2: **Simulated positions of equilibrium of dark pulses.** **a**, Evolution of a dark pulse with photon injection. At $T = 1$, photons with phase $\theta_n = 0$ are coupled to the 3rd mode relative to the pump. **b**, Temporal profiles of the dark pulse at the initial and final stages of the evolution. The net gain of the SWs calculated by Eq. S21 is plotted in blue. The SWs are stabilized to the predicted positions of equilibrium at the end of the evolution. The device parameters used in the simulations are $d_2 = -0.001$, $g/2\pi = 44.5$ Hz, $\kappa/2\pi = 579$ MHz, and the pumping parameters are $\zeta = 5$, $f = 2.536$.

where ϕ_L represents the location of LSW. By defining $\int_{-(D+W)}^{-W} \psi_{L0}^* d\phi$ as $Pe^{i\varphi_P}$, the net gain for LSW reads

$$G'_L = 2\epsilon_n P \cos(n\phi_L + \theta_n + \varphi_P). \tag{S18}$$

Until here the profiles of SWs are assumed unchanged. However, at time scales longer than the photon lifetime, modifications to the profiles of SWs should be considered. Interestingly, the lack of a general analytical solution for the LLE does not forbid the derivation of the positions of equilibrium for the SWs. We find that, for ϕ_L satisfying $\cos(n\phi_L + \theta_n) = 0$, the modified LSW

$$\psi_L(\phi) = \psi_{L0} e^{i\epsilon_n \sin(n\phi_L+\theta_n)/f} \tag{S19}$$

is a solution to Eq. S14 up to the first order. Furthermore, the net gain of the LSW at this location reads

$$G_L = \int_{-(D+W)}^{-W} (-2|\psi_L|^2 + f e^{i\epsilon_n \sin(n\phi_L+\theta_n)/f} \psi_L^* + f e^{-i\epsilon_n \sin(n\phi_L+\theta_n)/f} \psi_L) d\phi = 0. \tag{S20}$$

The vanishing gain shows that this ϕ_L corresponds to positions of equilibrium for the SWs, which are confirmed through numerical simulations in Fig. S2.

Based on the analysis above we anticipate that the net gain of the LSW along the microresonator could be modified to

$$G_L = 2\epsilon_n P \cos\varphi_P \cos(n\phi_L + \theta_n). \tag{S21}$$

Similarly for the RSW, the gain is $2\epsilon_n P \cos\varphi_P \cos(n\phi_R + \theta_n)$. The velocities of SWs can be thus expressed as

$$v_L = -\frac{G_L}{|\rho_H|^2 - |\rho_L|^2} = -\frac{2P\epsilon_n \cos\varphi_P}{|\rho_H|^2 - |\rho_L|^2} \cos(n\phi_L + \theta_n), \tag{S22}$$

$$v_R = \frac{G_R}{|\rho_H|^2 - |\rho_L|^2} = \frac{2P\epsilon_n \cos\varphi_P}{|\rho_H|^2 - |\rho_L|^2} \cos(n\phi_R + \theta_n). \tag{S23}$$

Here positive velocities indicate moving directions to the right. Combining the velocities of the two SWs gives the velocity of the dark pulse and the growth rate of the upper level as

$$v_{\rm dp} = \frac{\partial(\phi_R + \phi_L)/2}{\partial\tau} = \frac{v_R + v_L}{2} = -\frac{2P\epsilon_n \cos\varphi_P}{|\rho_H|^2 - |\rho_L|^2} \sin(n\pi\Lambda)\sin(n\phi_c + \theta_n), \tag{S24}$$

$$v_{\mathrm{r}}=\frac{\partial(\phi_R-\phi_L)/2}{\partial\tau}=\frac{v_R-v_L}{2}=\frac{2P\epsilon_n\cos\varphi_P}{|\rho_H|^2-|\rho_L|^2}\cos(n\pi\Lambda)\cos(n\phi_c+\theta_n), \tag{S25}$$

where $\phi_c=\frac{\phi_R+\phi_L}{2}$ represents the location of the energy center of the dark pulse and $\Lambda=(\phi_R-\phi_L)/2\pi$ is the duty cycle. Averaging Eq. S24 and S25 over the random phases of the stochastic photons (θ_n) yield the fluctuations of v_{dp} and v_r, which are also the responsivity of jitter- and breathe-type motions of the dark pulses to noises. The results are given by

$$\begin{aligned}\chi_{\mathrm{jitter}}=<v_{\mathrm{dp}}^2>/\epsilon_n^2&=\frac{4P^2\cos^2\varphi_P}{(|\rho_H|^2-|\rho_L|^2)^2}\sin^2(n\pi\Lambda)\frac{1}{2\pi}\int_{-\pi}^{\pi}\sin^2(n\phi_c+\theta_n)d\theta_n.\\&=\frac{P^2\cos^2\varphi_P}{(|\rho_H|^2-|\rho_L|^2)^2}[1-\cos(2\pi n\Lambda)],\end{aligned} \tag{S26}$$

$$\begin{aligned}\chi_{\mathrm{breathe}}=<v_{\mathrm{r}}^2>/\epsilon_n^2&=\frac{4P^2\cos^2\varphi_P}{(|\rho_H|^2-|\rho_L|^2)^2}\cos^2(n\pi\Lambda)\frac{1}{2\pi}\int_{-\pi}^{\pi}\cos^2(n\phi_c+\theta_n)d\theta_n.\\&=\frac{P^2\cos^2\varphi_P}{(|\rho_H|^2-|\rho_L|^2)^2}[1+\cos(2\pi n\Lambda)].\end{aligned} \tag{S27}$$

These are the complete versions of Eq. 4 and Eq. 5 in the main text.

D. Simulation of noise responsivity

Numerical simulations of the noise responsivity are performed as follows. To avoid the digitizing error in the simulation, instead of a stochastic force, we add a periodically modulated force to the nth mode[5], which can be expressed as:

$$\tilde{F}_n(\tau)=F_n e^{i\omega_m\tau} \tag{S28}$$

F_n is the amplitude of modulated force and ω_m is the modulation frequency. In the simulation we set $F_m=\sqrt{\frac{2g}{\kappa\Delta\tau}}$ and $\omega_m=0.1\times2\pi$. As expected, the motion of the dark pulses is also modulated at frequency ω_m. The intensity of the modulation peaks observed in the power spectral density of the central location and total energy of the dark pulse energy is normalized by $|F_n|^2$ to give the responsivity of jitter and total pulse energy to the applied force. The responsivity of breathe-type motion is further converted from the responsivity of energy by

$$\chi_{\mathrm{breathe}}=\frac{\chi_{\mathrm{energy}}}{4(|\rho_H|^2-|\rho_L|^2)^2}. \tag{S29}$$

We present simulated results in Fig. S3, in which systems with and without AMX are discussed. In the systems with AMX, we change the detuning values ζ from 4 to 14 to get dark pulses with different duty cycles. Due to the degenerate character of the dark pulses in the systems without AMX[6], we used square waves with different duty cycles to seed dark pulses with different duty cycles but with the same pumping parameters. The periodic dependence of the jitter responsivity is observed in both cases (Fig. S3b). The period is extracted via DFT and is plotted versus the duty cycle. Their relation matches well with Eq. S26.

E. Scaling law and comparison between bright solitons and dark pulses

In this section, we explore the scaling law of quantum timing jitter in bright solitons and dark pulses. Using $\theta=\phi/\sqrt{|d_2|}$, we further normalized Eq. (1) in the main text to

$$\frac{\partial\psi}{\partial\tau}=i\mathrm{sgn}(d_2)\frac{\partial^2\psi}{\partial\theta^2}+i|\psi|^2\psi-(1+i\zeta)\psi+f+\bar{\epsilon}(\theta,\tau), \tag{S30}$$

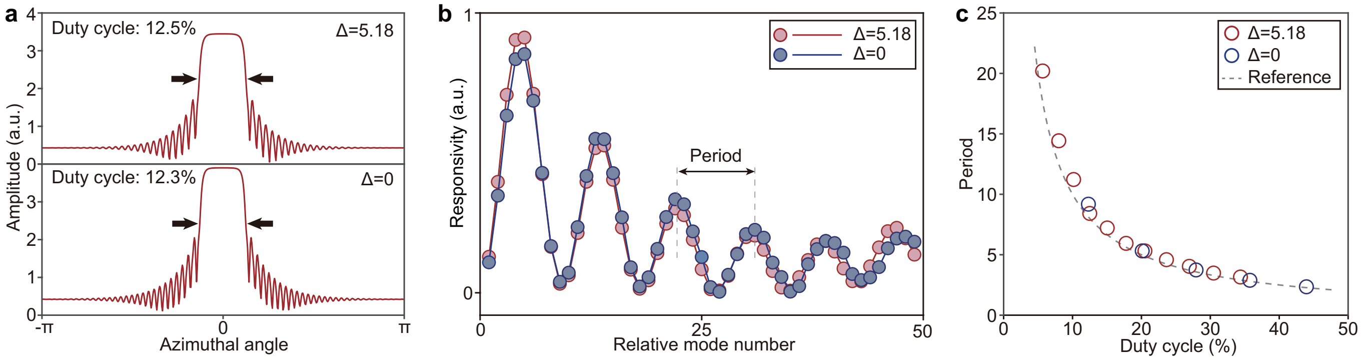

FIG. S3: **Simulation of noise responsivity.** **a**, Numerically simulated temporal profiles of the dark pulse with similar duty cycle generated in the systems with and without AMX. **b**, Numerically simulated jitter responsivity for the dark pulse in **a**. **c**, The relation of jitter responsivity modulation period with dark pulse duty cycle. The gray dotted curve is a reference line plotted as a y=1/x function. The device parameters used in the simulations are $d_2 = -0.0032$, $g/2\pi = 44.5$ Hz, and $\kappa/2\pi = 585$ MHz. Pumping parameters for the systems with AMX are $f = 4.09$, $\zeta = 11$ in **a** and **b**, ζ varying from 4 to 14 in **c**. Pumping parameters for the systems without AMX are $f = 5.86$, $\zeta = 14.093$.

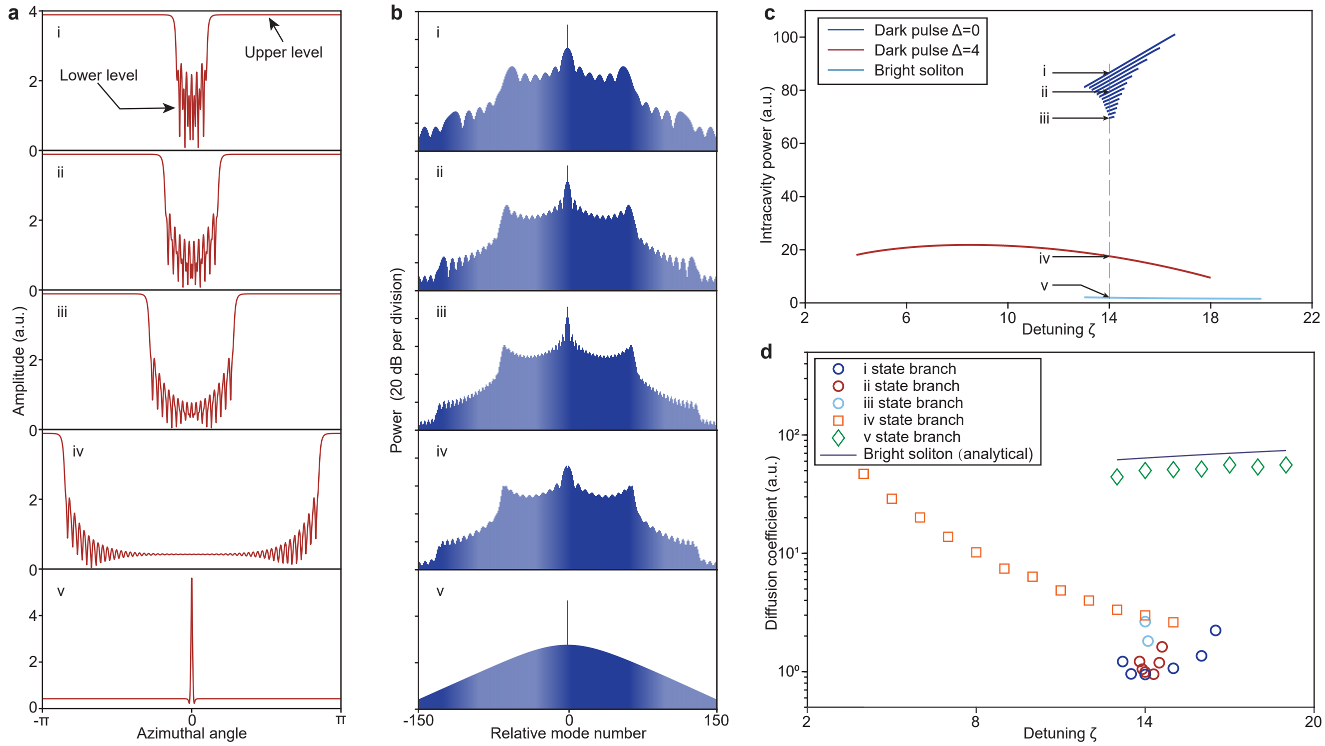

FIG. S4: **Noise comparison between bright solitons and dark pulses.** **a**, Numerically simulated temporal profiles for different dark pulse states and single bright soliton. The above three dark pulses are in the systems without AMX and the fourth is in the systems with AMX. The bright soliton is simulated at the same detuning value and absolute d_2 value as the dark pulse. All of these states are labeled in **c**. **b**, Corresponding optical spectra of **a**. **c**, Simulated intracavity power of different dark pulse states and bright soliton. **d**, Diffusion coefficient for bright soliton and dark pulse at different states. The parameters used in the simulation are: $f = 5.86$, $|d_2| = 0.0032$, $g/2\pi = 44.5$ Hz, $\kappa/2\pi = 585$ MHz.

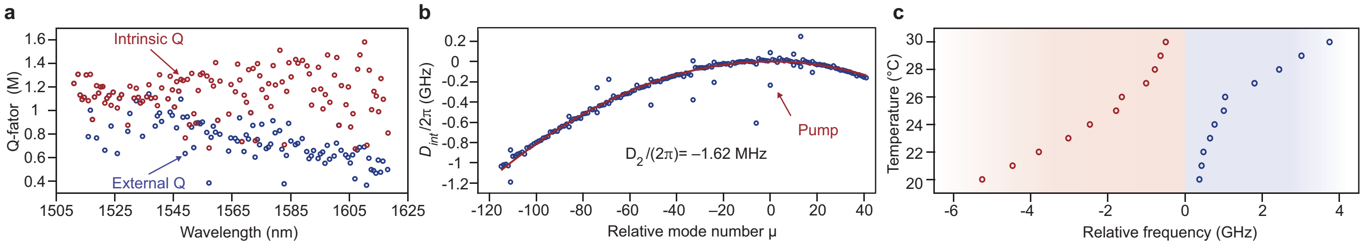

FIG. S5: **Q-factors, dispersion and thermal tuning of the microresonator. a,** Intrinsic and external Q factors of the comb-forming mode family. **b,** Measured mode family dispersion of the microresonator (blue) with the fitting parabola (red). The mode that is pumped for comb generation is indicated by the red arrow. **c,** Frequency deviation of pump mode (red) and auxiliary mode (blue) from the fitted line in **b**.

in which

$$\langle \bar{\epsilon}(\theta,\tau)\bar{\epsilon}^*(\theta',\tau')\rangle = \frac{2\hbar\omega_o^2 n_2 D_1}{\kappa n_o A_{\rm eff}\sqrt{|d_2|}}\delta(\theta-\theta')\delta(\tau-\tau'). \tag{S31}$$

sgn means taking the sign of d_2. In this frame, the waveforms of DKSs are solely dependent on the pumping conditions (ζ and f) and the sign of d_2. Therefore, we have

$$< \frac{\partial\theta_c}{\partial\tau}(\tau)\frac{\partial\theta_c}{\partial\tau}(\tau') >= \alpha'(\zeta, f, {\rm sgn}(d_2))\frac{2\hbar\omega_o^2 n_2 D_1}{\kappa n_o A_{\rm eff}\sqrt{|d_2|}}\delta(\tau-\tau'), \tag{S32}$$

where θ_c is the pulse central location. The PSD of θ_c takes the form

$$S_{\theta_c}(\omega) = \frac{2\alpha'(\zeta, f, {\rm sgn}(d_2))\hbar\omega_o^2 n_2 D_1}{\omega^2 n_o A_{\rm eff}\sqrt{|d_2|}}, \tag{S33}$$

which is converted to the PSD of timing jitter by

$$S_t(\omega) = \frac{2\alpha'(\zeta, f, {\rm sgn}(d_2))\hbar\omega_o^2 n_2\sqrt{|d_2|}}{\omega^2 n_o A_{\rm eff} D_1}. \tag{S34}$$

The diffusion coefficient is given by

$$D = \frac{2\alpha'(\zeta, f, {\rm sgn}(d_2))\hbar\omega_o^2 n_2\sqrt{|d_2|}}{3 n_o A_{\rm eff} D_1} = \alpha(\zeta, f, , {\rm sgn}(d_2))\frac{\sqrt{|d_2|} n_2}{n_o A_{\rm eff} D_1}, \tag{S35}$$

with $\alpha(\zeta, f, {\rm sgn}(d_2)) = 2\alpha'(\zeta, f, {\rm sgn}(d_2))\hbar\omega_o^2/3$. Note that according to the analytical results in Ref.[7], the alpha factor for bright soliton reads $\alpha = \frac{\hbar\omega_o^2\sqrt{\zeta}}{18}$.

In the main text, we have discussed the quantum decoherence of bright solitons and dark pulses by comparing a bright soliton and a dark pulse simulated using identical parameters except for their opposite dispersion. Here we investigate this issue with a broader selection of parameters. The results are summarized in Fig. S4. For dark pulses, we consider both cases with and without AMX. It is noted that without mode crossings the dark pulses are degenerate[6] in certain detuning ranges, and such degeneracy is lifted when AMX is introduced. The diffusion coefficients of bright solitons and dark pulses are weakly dependent on the detuning; however, the noise decreases rapidly with increased detunings for dark pulses existing in systems with AMX. For sufficiently-large detuning ($\zeta > 10$), the dark pulses generally show at least 10 dB better coherence than bright solitons.

II. ADDITIONAL EXPERIMENT

The quality factors of the mode family are summarized in Fig. S5a. The dispersion of the microresonator is measured using a widely-tunable laser with a wavelength calibrated by a fiber Mach-Zehnder interferometer. The resonant frequency of the μth mode relative to the pump, ω_μ, is presented in the form of integrated dispersion as defined by $D_{\rm int}$=ω_μ-ω_0-$D_1\mu$. The mode family shown in Fig. S5b is fitted using a parabola to give the chromatic

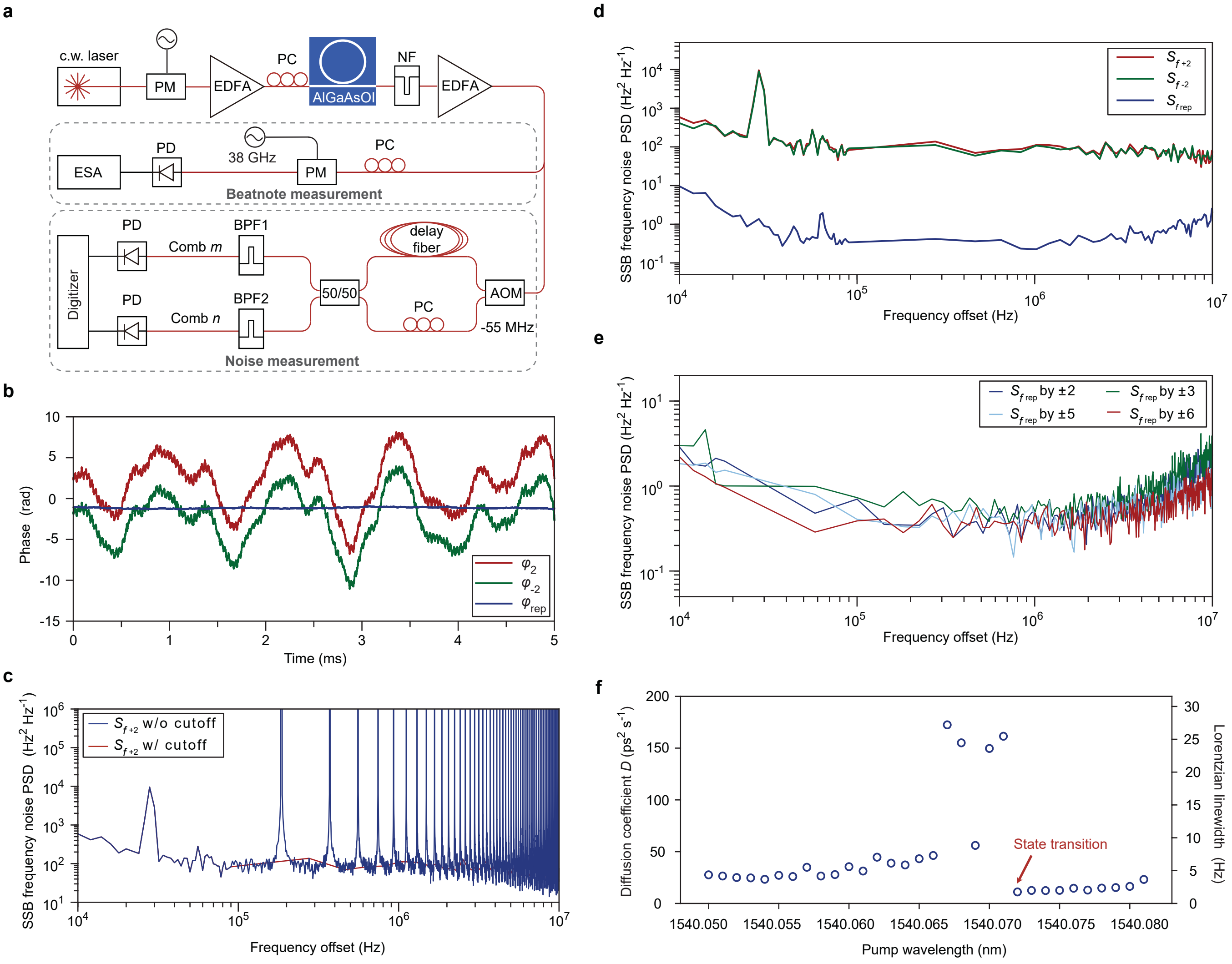

FIG. S6: **Experiment setup for dark pulse generation and noise characterization. a,** Experiment setup. PM: phase modulator; EDFA: erbium-doped-fiber-amplifier; PC: polarization controller; NF: fiber-Bragg-grating notch filter; AOM: acousto-optic modulator; BPF: fiber-Bragg-grating bandpass filter; PD: photodetector; ESA: electrical spectral analyzer. **b,** Phase diffusion of the ± 2 comb lines and repetition. Their relative phases are indicated by red color, green color, and blue. **c,** Single-sideband (SSB) frequency noise spectra of +2 comb lines w/ and w/o cutoff. **d,** Single-sideband (SSB) frequency noise spectra of detected comb lines and derived repetition rate. **e,** Single-sideband (SSB) frequency noise spectra of repetition rate derived by different comb lines. **f,** Diffusion coefficients and Lorentzian linewidth of the repetition rate as at different pump wavelengths.

dispersion as $D_2/2\pi = -1.63$ MHz. The pump mode is shifted from the fitted line as a result of AMX, which aids the formation of the dark pulse. The amount of frequency shift can be tuned by changing the temperature of the microresonator using a thermoelectric cooler. As shown in Fig. S5a, the red-shift is 0.4 GHz at 30 °C and 5.2 GHz at 20 °C. We select 23.9 °C as the temperature for comb generation.

The experimental setup for dark pulse generation and characterization is depicted in Fig. S6a. A phase modulator is inserted between the pump laser and the microresonator to create sidebands for noise calibration. The 91.5 GHz beatnote of the comb lines is measured by phase modulating the dark pulses at 38 GHz rate, and are then directed to a fast photodetector for analyzing the electrical spectra. Other experimental details have been mentioned in the main text.

The phase evolution $\phi_{m(n)}(t+\tau)-\phi_{m(n)}(t)$ is retrieved by applying Hilbert transformation to the trace recorded by the oscilloscope and subtracting the cumulative phase induced by the carrier frequency. Figure S6b shows the phase evolution of the $\pm$2nd comb lines, which are highly correlated over the timescale of measurement. After subtracting the common noise, the phase of the repetition frequency appears more stable than those of individual comb lines.

Apparently, these comb lines inherit the phase noise of the pump laser. The single-sideband frequency noise power spectral density of the comb line is given by

$$S_{f_m}(f) = \frac{PSD[\phi_{m(n)}(t+\tau) - \phi_{m(n)}(t)]}{|e^{-i2\pi f\tau} - 1|^2}. \tag{S36}$$

Here PSD denotes the power spectral density. τ is a delay between two arms of the interferometer. The singularity of the above equation at offset frequency where $|e^{-i2\pi f\tau} - 1|^2$ vanishes gives rise to the fringes in Fig. S6c. Therefore, we introduce a frequency cut-off at $1/\tau$ with N, beyond which only data points at $(N + 1/2)/\tau$ are plotted.

Figure S6d shows that the frequency noise of repetition rate is over two orders of magnitude lower than those of $\pm$2nd comb lines. In order to verify the repetition rate is independent of the selected comb line pair, four pairs of comb lines ($\pm$2, $\pm$3, $\pm$5 and $\pm$6) are selected to perform the same measurement, and the results are plotted in Fig. S6e. Clearly, these measurements give similar noise levels. We also measure diffusion coefficients at different pump wavelengths. When the pump laser is tuned to wavelengths longer than 1540.072 nm, a low-noise state appears (Fig. S6f), which is confirmed limited by quantum fluctuations in the main text.

[1] Chembo, Y. K. & Menyuk, C. R. Spatiotemporal lugiato-lefever formalism for Kerr-comb generation in whispering-gallery-mode resonators. *Phys. Rev. A* **87**, 053852 (2013).

[2] Paschotta, R. Noise of mode-locked lasers (part i): numerical model. *Appl. Phys. B* **79**, 153–162 (2004).

[3] Parra-Rivas, P., Gomila, D., Knobloch, E., Coen, S. & Gelens, L. Origin and stability of dark pulse Kerr combs in normal dispersion resonators. *Opt. Lett.* **41**, 2402–2405 (2016).

[4] Wang, H. *et al.* Self-regulating soliton switching waves in microresonators. *Phys. Rev. A* **106**, 053508 (2022).

[5] Matsko, A. B. & Maleki, L. Noise conversion in Kerr comb rf photonic oscillators. *J. Opt. Soc. Am. B* **32**, 232–240 (2015).

[6] Lobanov, V., Lihachev, G., Kippenberg, T. & Gorodetsky, M. Frequency combs and platicons in optical microresonators with normal GVD. *Opt. Express* **23**, 7713–7721 (2015).

[7] Matsko, A. B. & Maleki, L. On timing jitter of mode locked kerr frequency combs. *Opt. Express* **21**, 28862–28876 (2013).
